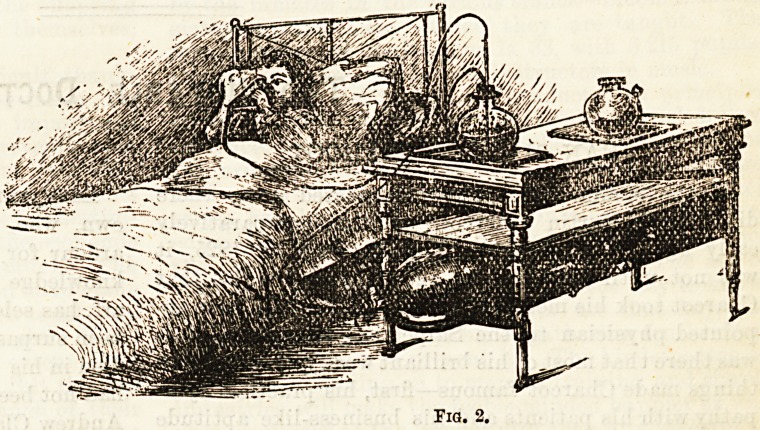# Modes of Administering Oxygen

**Published:** 1894-01-06

**Authors:** Benjamin Ward Richardson


### MODES OF ADMINISTERING OXYGEN.

By Sir BENJAMIN WARD RICHARDSON, M.D., F.R.S.

THE best mode of administering oxygen gas in cases of disease has been imperfectly studied. I have paid some attention to this subject, and have tried every method, I believe, that has been suggested without arriving at the simplicity and practical success that is desirable. The two or three methods I shall be content in describing are useful, but it must be understood that I do not claim for them that they are perfect. On the contrary, they admit of much improvement. They have one advantage, however, that they bring the remedy within the reach of every practitioner.

Originally I made the oxygen in the usual way from chlorate of potash, collecting the gas in a large holder, washing it well, and then exhausting it through a tap attached to a long tube ending in a mouthpiece and allowing the gas that was drawn from the holder to be replaced by water. The plan was too troublesome and too cumbersome to be adapted to the necessities of practice.

I moved from this method to another one much more convenient. I took advantage of peroxide of hydrogen and permanganate of potassa to effect the object in view When solution of hydrogen peroxide of ten-volume strength has added to it solution of permanganate of potassa, both substances yield their oxygen and a free supply of oxygen can be evolved. The liquid, during the process, loses its deep red colour, becoming quite colourless when action ceases.

The following apparatus is devised for this mode of

FIG. 1.

administration: A glass globe, Fig. 1, capable of holding a pint of solution, is furnished with two necks—one neck standing out straight at the top, the other standing out obliquely. Into the straight neck of this vessel there is inserted the neck of another globe with a tap intervening, as shown in the drawing. Into the oblique neck is inserted the end of a long flexible tube three-quarters-of-an-inch in diameter, which tube terminates at the free end in a cellulite double-valved mouthpiece, which adapts itself to the face of the patient, and can be washed as if it were made of glass. Into the lower globe is placed the solution of peroxide, into the upper one, with the tap closed, the solution of permanganate. The patient fits the mouthpiece closely over the mouth and nostrils, and without removing it commences to breathe from it. At the same time the tap is slowly turned, and the permanganate solution is allowed to run into the oxygen solution. There is soon yielded an abundant supply of oxygen gas, and with little trouble inhalation can be maintained for any length of time on repetition of the solutions as they wear out.

In another plan, depicted in the second figure, advantage has been taken of the compressed oxygen supplied in strong iron bottles by Messrs. Brin. For

FIG. 2.

hospital use I have devised a table which moves easily on wheels from bed to bed, so that inhalation can be used in the course of the day by several patients following each other. The table carries on its upper surface one or two jars of the same kind as the lower jars in the previously named apparatus, but in this case the upper glass globe for permanganate is dispensed with. The compressed oxygen bottle is placed under the table on a shelf which supports it, the part of the bottle with tap standing a little out at one end, as shown in the drawing. The tube and mouthpiece are the same, but one end of the tube is attached to the neck of the iron bottle holding the oxygen. The glass tube running into the glass jar proceeds nearly to the bottom, and the jar itself is half filled with pure water. The table brought to the side of the bed, the inhaling tube attached at one end to the other neck of the jar is applied to the mouth and nostrils of the patient by the mouthpiece, and the tap of the iron bottle is gently turned until bubbles of gas escape freely through the water. As before, the patient can continue to breathe without removing the mouthpiece, and the inhalation can go on so long as it may be considered necessary. In the drawing (Fig. 2) the artist has depicted the apparatus as it is used in the London Temperance Hospital, the patient being a young girl under my care there suffering from cyanosis owing to patency of the foramen ovale in the auricular septum of the heart.

The quality of the oxygen supplied by this method is neutral. It can be modified in various ways by modification of the fluid in the jar or globe on the table. The water in the globe can be made cold by the addition of ice; or it can be made hot by the addition of heated water. It can be ozonised by allowing ozonic ether to float on the surface of the water, and it can be made hypnotic by floating anhydrous ether on the water. It can be made negative by the addition of ammonia to the water. By this simple means I have been able to make oxygen the bearer or menstruum of several medicinal substances, and to supply for inhalation a variety of atmospheres. The following are useful formulæ:—

*Pure sulphuric ether* in the proportion of two fluid ounces on a pint of water, at 60 deg. Fahr., yields, with the oxygen current, a gentle hypnotic atmosphere.

*Ozonic ether* in the proportion of one ounce on the pint of water, yields an ozonic atmosphere which is very useful in cases of ulcerated fauces and fœtid breath; also in cases of phthisis with cavities.

*Ammonia* in the proportion of one fluid drachm, mixed with the pint of water, at 60 deg. Fahr., yields an atmosphere that can be employed with advantage in febrile states, and in conditions where precipitation of fibrine in the right cavities of the heart is threatened.

*Spirit of turpentine* floated on the water in the proportion of half a fluid ounce to one pint of water, yields an atmosphere of good account in hæmorrhagic cases, and where the diuretic effect of the medicine is required.

## Figures and Tables

**Fig. 1. f1:**
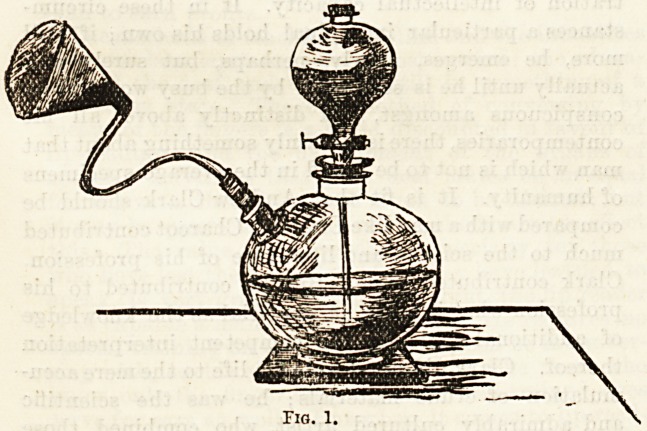


**Fig. 2. f2:**